# Leukocyte telomere length and circulating MiRNAs in relation to cardiovascular outcomes in older adults

**DOI:** 10.1186/s12877-026-07042-4

**Published:** 2026-02-02

**Authors:** Rossella La Grotta, Paolina Crocco, Aleksandra Leonova, Salvatore Claudio Cosimo, Francesco Morelli, Serena Dato, Giuseppe Passarino, Giuseppina Rose

**Affiliations:** 1 Department of Biology, Ecology and Earth Sciences, University of Calabria, Rende, CS 87036 Italy; 2 SADEL S.p.A, Cotronei, Italy

**Keywords:** Telomere length, MicroRNAs, Cardiovascular disease, Atrial fibrillation, Heart failure, Stroke, Aging, Biomarkers

## Abstract

**Background:**

Telomere shortening and circulating microRNAs (miRNAs) are recognized molecular hallmarks of biological aging. Both have been implicated in cardiovascular disease (CVD), yet their potential interplay and combined contribution to cardiovascular vulnerability, and potential clinical relevance in older adults remain unclear.

**Methods:**

We investigated the associations of leukocyte telomere length (LTL) and five circulating miRNAs (miR-21-5p, miR-23a-3p, miR-34a-5p, miR-92a-3p, and miR-486-5p), previously linked to telomere maintenance and cardiovascular pathology, with CVD outcomes in a cohort of 624 elderly individuals (aged 60–98 years). LTL was measured in all participants, while miRNAs were quantified in a subset of 210 individuals. Logistic regression analyses adjusted for demographic and clinical covariates were applied to assess associations of LTL and miRNAs with overall CVD and specific conditions, including atrial fibrillation (AF), heart failure (HF), ischemic cardiomyopathy (ICM), and stroke.

**Results:**

Shorter LTL was independently associated with higher AF risk (OR = 0.25, 95% CI 0.11–0.65, *p* = 0.004) but not with other CVD outcomes. In AF, miR-23a-3p and miR-92a-3p were not individually associated with risk but showed opposite directions of association when included together in fully adjusted models (miR-23a-3p protective; miR-92a-3p risk-enhancing), independently of LTL. Lower miR-92a-3p levels were also associated with stroke risk, whereas associations of miR-34a-5p with HF and miR-486-5p with ICM were attenuated after adjustment. No significant correlations were found between LTL and circulating miRNAs.

**Conclusions:**

Shorter telomeres and distinct expression patterns of the analysed miRNAs were independently associated with specific cardiovascular outcomes in older adults, suggesting that these biomarkers reflect complementary molecular aspects of cardiovascular vulnerability in aging. While their addition to conventional risk factors did not significantly improve risk discrimination, they may provide mechanistic insights into biological processes underlying cardiovascular risk in aging populations.

**Supplementary Information:**

The online version contains supplementary material available at 10.1186/s12877-026-07042-4.

## Background

 Cardiovascular diseases (CVDs), a heterogeneous group of disorders affecting the heart and blood vessels, remain the leading cause of morbidity and mortality worldwide. Recent global estimates indicate that the burden of CVDs has continued to increase through 2023 and is projected to rise further in the coming decades, largely driven by population aging and the growing prevalence of cardiometabolic risk factors such as obesity, diabetes, and hypertension [1, 2].

CVDs arise from complex interactions among genetic, environmental, and lifestyle factors. Despite advances in elucidating their molecular mechanisms, the identification of reliable biomarkers for early detection and risk stratification remains a major goal in translational research. In this context, telomeres and microRNAs (miRNAs) have emerged as promising candidates for assessing biological aging and cardiovascular vulnerability.

Telomeres are repetitive DNA-protein structures that protect chromosome ends and preserve genomic stability [3]. Telomere length (TL) progressively shortens with cell division and age, a process accelerated by oxidative stress and chronic inflammation, making it a hallmark of cellular aging [4, 5]. Findings suggest that telomere attrition reflects systemic biological aging. Accordingly, shorter leukocyte telomere length (LTL) has been consistently associated with age-related diseases, including diabetes, Alzheimer’s, and chronic kidney disease, as well as increased mortality and reduced lifespan [6–9]. Moreover, epidemiological evidence suggests that shorter telomeres are associated with higher cardiovascular risk [10–13], suggesting a potential role for TL in risk stratification.

At the same time, miRNAs, small, non-coding RNAs that regulate gene expression post-transcriptionally, are increasingly recognized as key epigenetic regulators in cardiovascular physiology and pathology [14, 15]. They play critical roles in endothelial dysfunction, inflammation, fibrosis, and cardiac remodeling, which are central mechanisms in the onset and progression of CVDs [16–19].

Emerging evidence points to a bidirectional relationship between telomere dynamics and miRNA regulation. Certain miRNAs can influence telomere maintenance by targeting telomere- or telomerase-associated genes, while telomere dysfunction can, in turn, alter miRNA expression profiles [20, 21]. Some miRNAs promote telomere shortening, whereas others contribute to telomere stabilization [22, 23].

Building on this link, we investigated the interplay between LTL and circulating miRNAs in relation to cardiovascular outcomes, including atrial fibrillation (AF), heart failure (HF), ischemic cardiomyopathy (ICM), and stroke, in older adults. LTL was analyzed in a cohort of 624 participants (aged 60–98 years) and circulating levels of five miRNAs (miR-21-5p, miR-23a-3p, miR-34a-5p, miR-92a-3p, and miR-486-5p), previously associated with telomere regulation and cardiovascular disease, were measured in a subset of 210 participants with available plasma samples.

Among these, miR-21-5p, miR-23a-3p, and miR-34a-5p are of particular interest as potential molecular mediators linking telomere dysfunction and CVD.

miR-21-5p is involved in cardiac fibrosis, inflammation, and apoptosis, and is upregulated in various myocardial disorders [24, 25]. It has also been linked to telomere regulation, as telomerase reverse transcriptase (TERT) silencing reduces both telomerase activity and miR-21 expression [26], whereas miR-21 itself can modulate TERT expression [27, 28].

miR-23a-3p regulates cardiomyocyte apoptosis [29] and contributes to coronary artery disease progression [30, 31]. It can directly affect telomere integrity by downregulating telomeric repeat binding factor 2 (TRF2), a key component of the shelterin complex responsible for telomere protection [32].

miR-34a-5p is a well-established senescence-associated miRNA involved in vascular aging, fibrosis, and cardiovascular dysfunction [33, 34]. It also suppresses TERT’s expression and inhibits telomerase activity, promoting telomere erosion and cellular senescence [35].

Although miR-92a-3p and miR-486-5p are not directly linked to telomere biology, they are strongly implicated in CVD pathophysiology and may offer complementary insights into disease mechanisms.

miR-92a-3p influences neovascularization, endothelial function, and ischemic injury [36–38], while miR-486-5p contributes to cardiac remodeling and ischemic protection [39–41].

Our aim was to clarify the independent and combined contributions of telomere attrition and miRNA dysregulation in cardiovascular vulnerability in aging, with potential implications for understanding the molecular basis of cardiovascular aging and improving biomarker-based risk assessment.

## Methods

### Study population

This retrospective study included a cohort of 624 individuals of Calabrian descent, were recruited from multiple nursing home residents (NHRs) across the Calabria region as part of regional campaigns aimed at monitoring the quality of aging.

The cohort comprised 232 individuals with a clinical diagnosis of cardiovascular disease (CVD) (mean age: 83.2 ± 7.08 years; 63.4% females). The CVD group included patients with atrial fibrillation (AF), heart failure (HF), ischemic cardiomyopathy (ICM), and stroke. A control group of 392 healthy individuals (mean age: 80.02 ± 7.96 years; 72.4% females) was also included.

Leukocyte Telomere Length (LTL) was measured in the entire cohort (*n* = 624). MiRNA levels were subsequently quantified in plasma samples from a subset of 210 individuals (100 CVD patients and 110 controls) for whom plasma was available.

The study was approved by the local Ethics Committee (Comitato Etico Regione Calabria–Sezione Area Nord, approval code n. 25/2017, dated October 31, 2017). All participants gave written informed consent in accordance with ethical standards and privacy regulations.

### Clinical and laboratory characterization of the study cohort

All participants underwent a comprehensive clinical and geriatric evaluation aimed at assessing cognitive function, physical health, functional status, and social conditions. Trained interviewers administered a structured questionnaire to collect relevant data. Information on demographic characteristics (age, sex) and anthropometric measurements (height, weight, and body mass index [BMI]) was recorded for every subject. The complete questionnaire is provided as Supplementary File 1.

Clinical data included systolic and diastolic blood pressure (SBP and DBP), medical history of hypertension and diabetes. CVD diagnosis was established based on thorough medical histories, clinical signs and symptoms, diagnostic imaging, and physical or laboratory assessments, following international guidelines and confirmed by a board-certified cardiologist.

Peripheral blood samples were obtained from all participants for laboratory analyses, including telomere length quantification, miRNA expression profiling, and evaluation of conventional biochemical biomarkers, performed according to standardized protocols. Conventional biomarkers, encompassing lipid profile, glycemic indices, renal function parameters, and inflammatory markers, are presented in Table 1.

**Table 1:** Baseline characteristics of the study population stratified by cardiovascular disease (CVD) status

	CVD		
Variable	Absent (*n* = 392)	Present (*n* = 232)	*p*-value^*^
Age (years)	80.02 ± 7.96	83.20 ± 7.08	< 0.001
Female sex, *n* (%)	72.4%	63.4%	0.022
BMI (kg/m²)	27.03 ± 6.25	27.11 ± 5.75	0.837
Total Cholesterol (mg/dL)	170.94 ± 40.55	152.40 ± 35.49	< 0.001
HDL Cholesterol (mg/dL)	53.48 ± 17.36	48.18 ± 12.61	< 0.001
LDL Cholesterol (mg/dL)	99.04 ± 32.18	82.98 ± 30.17	< 0.001
Triglycerides (mg/dL)	126.01 ± 69.85	116.39 ± 63.02	0.043
Fasting Glucose (mg/dL)	102.12 ± 41.97	100.94 ± 32.00	0.472
HbA1c (%)	6.09 ± 1.42	6.01 ± 1.56	0.222
DBP mean (mmHg)	74.68 ± 7.18	72.26 ± 8.17	0.001
SBP mean (mmHg)	126.61 ± 11.28	126.06 ± 13.04	0.627
Azotaemia (mg/dL)	45.54 ± 17.96	55.34 ± 31.60	0.002
Creatinine (mg/dL)	1.06 ± 0.36	1.17 ± 0.53	0.012
Uric Acid (mg/dL)	4.63 ± 1.33	5.13 ± 1.60	0.002
Albumin (g/L)	54.45 ± 6.29	53.14 ± 6.51	0.009
C-reactive Protein (mg/L)	10.12 ± 18.87	16.04 ± 28.88	0.015
Hypertension, yes (%)	67.6%	81.0%	< 0.001
Diabetes Mellitus, yes (%)	23.5%	29.7%	0.102
Atrial Fibrillation, yes (%)		26.7%	
Heart Failure, yes (%)		20.3%	
Ischemic Cardiopaty, yes (%)		60.8%	
Stroke, yes (%)		24.1%	

Continuous variables are presented as mean ± SD and categorical variables as percentage

*Abbreviations*: *BMI* Body Mass Index, *HDL* High Density Lipoprotein, *LDL* Low Density Lipoprotein, *HbA1c* Glycated Haemoglobin, *SBP* Systolic Blood Pressure, *DBP* Diastolic Blood Pressure

*P value from t-test or Mann–Whitney depending on continuous data distribution and Fisher’s exact test of association for categorical variables

### Leukocyte telomere length (LTL)

Following the isolation of genomic DNA from whole blood or buffy coats using the salting out process [42], LTL was measured using a previously modified quantitative real-time PCR protocol [43]. The T/S ratio was determined by comparing the copy number of telomeric repeats (T) to a single-copy gene (S) employed as an internal reference, to determine relative telomere length [44]. The reference gene was 36B4, which codes for the ribosomal acid phosphoprotein P0. For each sample, the telomeric (T) and 36B4 (S) sequences were amplified in triplicate. Two separate master mixes were prepared, containing SYBR Green and the T- or S-specific primers at the recommended concentrations; 15 µL of master mix and 5 µL of genomic DNA (3 ng/µL, or 15 ng total) were added to each well. Each plate included a sample of calibrator DNA (Roche, Milan, Italy; 5 µL at 3 ng/µL) and two standard curves (36B4 and telomeres) generated by serial dilutions (factor 1.68) of reference DNA (30 − 2 ng). Reactions were carried out on 96-well plates (Thermo Fisher Scientific, Waltham, MA, USA) with the QuantStudio 3 instrument (Applied Biosystems) and the following temperature profile: 95 °C for 10 s, then 30 cycles of 95 °C for 5 s, 57 °C for 15 s and 72 °C for 20 s. T. The results, normalized to the calibrator sample, were reported as the T/S ratio. As a quality control, more than 20% of the samples were retested on independent plates.

### Plasma MiRNA extraction and quantification

For miRNA expression analysis, plasma was obtained by centrifuging whole blood at 1800 × g for 10 min at room temperature. Before RNA extraction, hemolysis was assessed spectrophotometrically by measuring absorbance at 414 nm using a NanoDrop ND-1000 spectrophotometer (Thermo Fisher Scientific, Milan, Italy); no significant hemolysis was detected in the included samples. Plasma was then transferred to RNase-free tubes and subjected to a second centrifugation at 1200 × g for 20 min at 10 °C to remove residual cellular debris. Samples were aliquoted to avoid freeze–thaw cycles and stored at − 80 °C until RNA extraction.

Total RNA was isolated using the mirVana™ PARIS™ RNA and Native Protein Purification Kit (Cat. No. AM1556, Thermo Fisher Scientific), following the manufacturer’s instructions. Briefly, 200 µl of plasma was mixed with an equal volume of 2× denaturing solution. Subsequently, 25 fmol of synthetic cel-miR-39-3p (Thermo Fisher Scientific, Assay ID: 478293_mir) was added as a spike-in control. After organic extraction with acid-phenol and chloroform, 1.25 volumes of 100% ethanol (room temperature) were added to the aqueous phase. The RNA was washed three times and eluted in 100 µl of pre-warmed elution solution.

Complementary DNA (cDNA) was synthesized from 2 µl of eluted RNA using the TaqMan™ Advanced RNA cDNA Synthesis Kit (Cat. No. A25576, Applied Biosystems), according to the manufacturer’s two-step protocol. The process included poly(A) tailing, 5′-adapter ligation, reverse transcription, and universal pre-amplification using the primers provided in the kit. The resulting cDNA was stored at − 20 °C until quantitative PCR analysis.

Quantitative real-time PCR (qPCR) was performed using 5 µl of preamplified cDNA (diluted 1:10), 10 µl of TaqMan™ Fast Advanced Master Mix (without UNG, Cat. No. A44360), and 1 µl of TaqMan™ Advanced miRNA Assays specific for each target miRNA: hsa-miR-21-5p (ID: 477975), hsa-miR-23a-3p (ID: 478532), miR-34a-5p (ID: 478048), miR-92a-3p (ID: 477827) and miR-486-5p (ID: 478128). Reactions were run on a QuantStudio™ 3 Real-Time PCR System (Applied Biosystems, Milan, Italy) using the following cycling conditions: 40 cycles of 95 °C for 1 s (denaturation) and 60 °C for 20 s (annealing/extension).

Each sample was analysed in triplicate. Negative controls (no-template controls, NTCs) using nuclease-free water in place of the assay were included in each run. A cycle threshold (Ct) value < 35 was considered valid.

Hsa-miR-484 (ID: 478308) was selected as an endogenous control for qPCR data normalization based on prior evidence of its stability in plasma and serum [45]. In our cohort, miR-484 Ct values remained consistent regardless of age, sex, or across groups, further supporting its appropriateness as a reference gene. Relative miRNA expression levels were calculated using the 2^-ΔCt method [46] and log-transformed to approximate a normal distribution prior to statistical analysis.

### Statistical analysis

Quantitative variables are provided as medians and their interquartile range or means and their standard deviation depending on their distribution (Shapiro–Wilk test for normality). For qualitative variables, relative frequencies are given as percentages. Demographic and clinical variables were compared between groups using the Mann–Whitney or Student t tests according to the normality of the data for continuous variables and Fisher’s exact test for categorical variables.

LTL (expressed as mean T/S ratio) was natural log transformed. Correlation analyses between LTL and continuous variables were conducted using Pearson or Spearman correlation coefficients, depending on normality. Partial correlations controlling for age and sex were also performed.

Logistic regression models with age and sex as covariates were employed to evaluate whether LTL was associated with the presence of cardiovascular disease and specific subtypes. Additional models further adjusted for clinical and biochemical parameters that showed significant differences between groups or significant correlations with LTL, to account for potential confounding. Odds ratios (OR) and 95% confidence intervals (CI) were reported.

In a subset of participants with available plasma samples, we quantified selected circulating miRNAs. To assess potential selection bias, participants with available plasma samples for miRNA analysis (*n* = 210) were compared with those without available plasma samples (*n* = 414) with respect to demographic characteristics, cardiovascular risk factors, and disease prevalence.

Comparisons of miRNA levels (natural log transformed) between subjects with and without diagnosis of CVD were performed using logistic models adjusted for age and sex and additional relevant covariates.

The relationship between miRNA expression and LTL was assessed using Pearson or Spearman correlation coefficients, as well as partial correlations adjusted for age and sex. To explore the association of LTL and miRNA levels with the presence of cardiovascular disease, multivariable logistic regression models were applied, including LTL, individual miRNAs, and relevant covariates (age, sex, clinical parameters). Specifically, Model 1 included the individual miRNA and was adjusted for age and sex. Model 2 further adjusted for established cardiovascular risk factors (BMI, hypertension, diabetes mellitus, LDL-cholesterol, HDL-cholesterol, triglycerides, and creatinine). Model 3 evaluated each biomarker separately (i.e., one miRNA at a time and LTL) using the same covariate set as Model 2, to assess the independent association of each biomarker with the outcome. Model 4 included LTL and all miRNAs simultaneously, in addition to the same clinical covariates, to assess their mutual independence and their combined contribution to the outcome.

As a sensitivity analysis, we additionally adjusted Model 4 for inflammatory biomarkers (C-reactive protein and albumin) to evaluate whether associations were robust to further adjustment for systemic inflammation.

Receiver operating characteristic (ROC) curve analysis was performed to obtain area under the curve (AUC) values for evaluating diagnostic performance of LTL and each plasma miRNA for CVD.

All statistical tests were two-sided, and a *p*-value < 0.05 was considered statistically significant. Additionally, the false discovery rate (FDR) was applied to correct for multiple comparisons, with q-value < 0.05 set as the threshold for statistical significance [47]. Analyses were performed using R version 4.5.1 (2025-06-13 ucrt).

## Results

### Descriptive analysis of the study population

The study cohort consisted of 624 individuals, comprising 232 patients with cardiovascular disease (CVD) and 392 healthy controls. Demographic, clinical, and biochemical characteristics of both groups are summarized in Table 1.

Compared to controls, individuals with CVD were significantly older and less frequently female (*p* < 0.05). The CVD group exhibited a less favourable profile in markers of renal function and inflammation, showing significantly higher levels of azotaemia, creatinine, uric acid, and C-reactive protein, alongside lower albumin concentrations (*p* < 0.05 for all). Conversely, despite a higher prevalence of hypertension (81.0% vs. 67.6%, *p* < 0.001), CVD patients presented with lower mean levels of total cholesterol, LDL-cholesterol, triglycerides, HDL-cholesterol, and diastolic blood pressure (*p* < 0.05 for all), which may reflect prior therapeutic interventions.

The subsample used for miRNA analyses comprised 210 participants. To assess potential differences related to plasma sample availability, individuals with available plasma samples were compared with those without available plasma samples in terms of demographic characteristics, cardiovascular risk factors, biochemical parameters, and disease prevalence (Supplementary File 2). Participants with miRNA data were older and showed modest differences in lipid profile and overall CVD prevalence; however, no substantial differences were observed for most cardiovascular risk factors, renal and inflammatory markers, or for the distribution of individual CVD subtypes.

### Leukocyte telomere length and correlations with clinical parameters

To identify potential factors that may affect Leukocyte telomere length (LTL), we tested the correlations between this measure and demographic, clinical, and biochemical factors in the entire cohort. LTL was negatively correlated with age (sex-adjusted partial correlation: r_partial_ = -0.17, *p* < 0.0001). Furthermore, partial correlation analyses adjusted for age and sex revealed LTL associations with blood biomarkers of systemic inflammation. Specifically, LTL was positively correlated with albumin (r_partial_ = 0.13, *p* = 0.002) and negatively correlated with C-reactive Protein (r_partial_ = -0.12, *p* = 0.008).

These correlations remained similar when the two groups (CVD and Controls) were analysed separately.

### Leukocyte telomere length and risk of cardiovascular disease

Logistic regression analysis adjusted for age and sex showed no statistically significant difference in LTL between individuals with and without a general CVD diagnosis (*p* > 0.05).

Given the heterogeneity of the CVD group, we further explored LTL in relation to individual disease subtypes (Atrial Fibrillation [AF], Heart Failure [HF], Ischemic Cardiomyopathy [ICM], and Stroke).

**Fig. 1:**
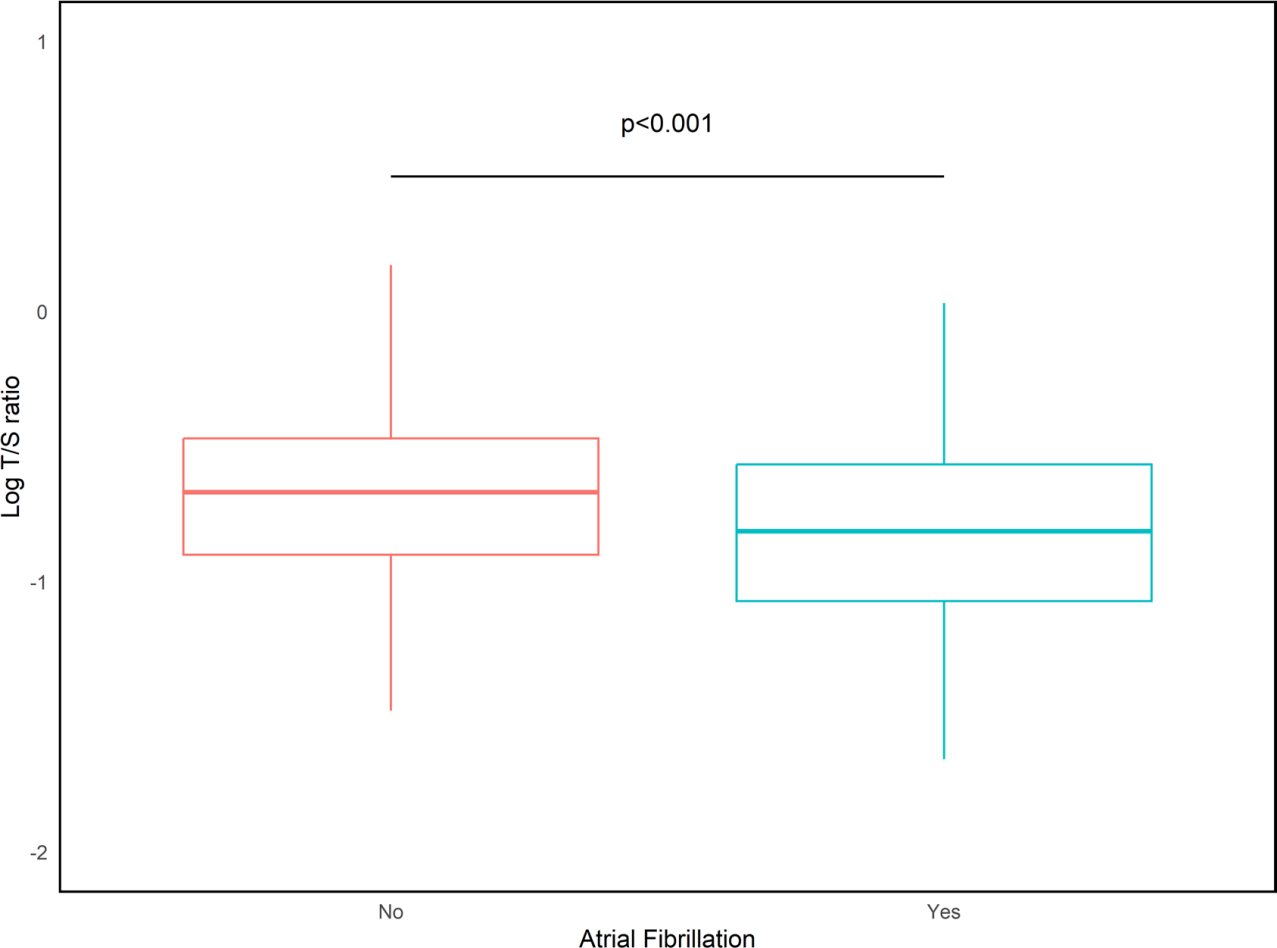
Distribution of leukocyte telomere length (LTL) (expressed as logT/S ratio) in patients with (Yes) and without (No) Atrial Fibrillation. *Note*: *p*-value indicates the significance of the difference after adjusting for age and sex

While no association was observed between LTL and the overall CVD status, we identified a strong, inverse association with AF. As shown in Fig. 1, shorter telomeres were associated with increased risk of AF (Odds Ratio [OR] = 0.311, 95% CI: 0.160–0.603; *p* < 0.001; qFDR = 0.0028). The robust association was confirmed in a final multiple logistic regression model, including, behind age and sex as covariates, also established CVD risk factors (BMI, hypertension, diabetes, HDL-cholesterol, LDL-cholesterol, triglycerides, and creatinine). In this model, longer LTL remained independently associated with a significantly reduced risk of AF (OR = 0.250, 95% CI: 0.11 − 0.651, *p* = 0.004; qFDR = 0.017).

### Circulating MiRNAs and risk of cardiovascular disease

In a subsample of 210 participants with available plasma, we measured the circulating levels of five miRNAs known for their potential role in telomere biology and cardiovascular risk, namely miR-21-5p, miR-23a-3p, miR-34a-5p, miR-92a-3p, and miR-486-5p. Additional descriptive characteristics of the stratified sample by presence or absence of CVD are provided in Supplementary File 3.

**Fig. 2:**
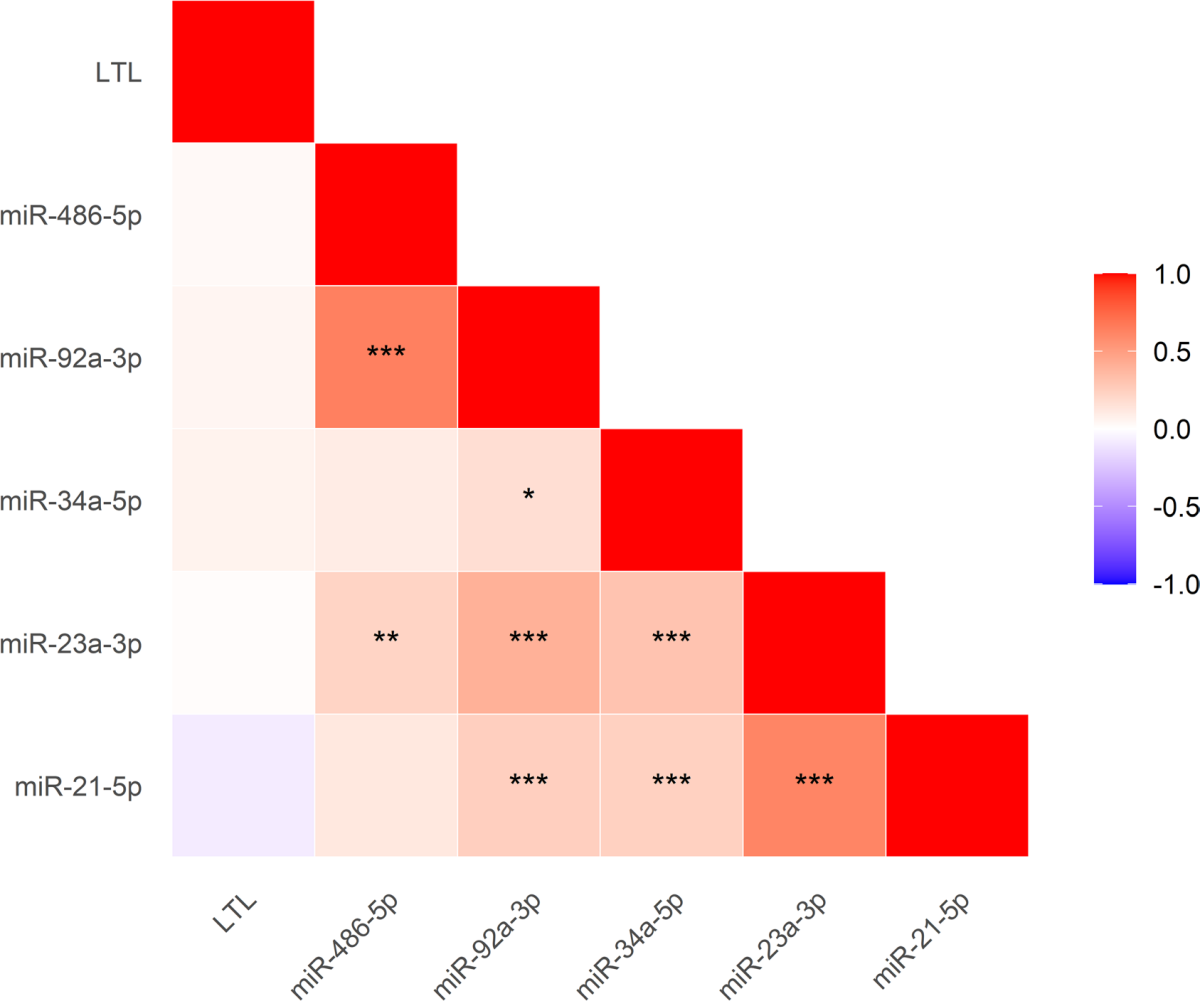
Correlation Matrix of miRNA Levels and Leukocyte Telomere Length (LTL). *Note*: The triangular matrix displays correlation among the expression levels of five microRNAs (miR-21-5p, miR-23a-3p, miR-34a-5p, miR-92a-3p, miR-486-5p) and Leukocyte Telomere Length (LTL). The color scale (side bar) indicates the value of the correlation coefficient, where red denotes a positive correlation (approaching + 1) and blue denotes a negative correlation (approaching − 1). Statistically significant correlations are marked as follows: ^*^p < 0.05, ^∗∗^*p* < 0.01, ^∗∗∗^*p* < 0.001

First, we evaluated the correlations between the miRNAs themselves, as well as between each miRNA and LTL. The results are summarized in the correlation matrix (Fig. 2). Overall, the analysis revealed strong positive correlations among the majority of the tested microRNAs. Crucially, no statistically significant correlations were found between LTL and the expression levels of any of the five tested miRNAs.

Initial analyses revealed a positive correlation between age and the expression levels of miR-21-5p and miR-23a-3p after correction for sex (r_partial_ =0.16, *p* = 0.02 and r_partial_ = 0.24, *p* < 0.001, respectively). Furthermore, adjusting for age and sex revealed positive correlations between miR-21-5p and markers of renal function (azotaemia: r_partial_ = 0.15, *p* = 0.035; creatinine: r_partial_ = 0.19, *p* = 0.006), while miR-486-5p positively correlated with albumin levels (r_partial_ =0.20, *p* = 0.004).

**Fig. 3:**
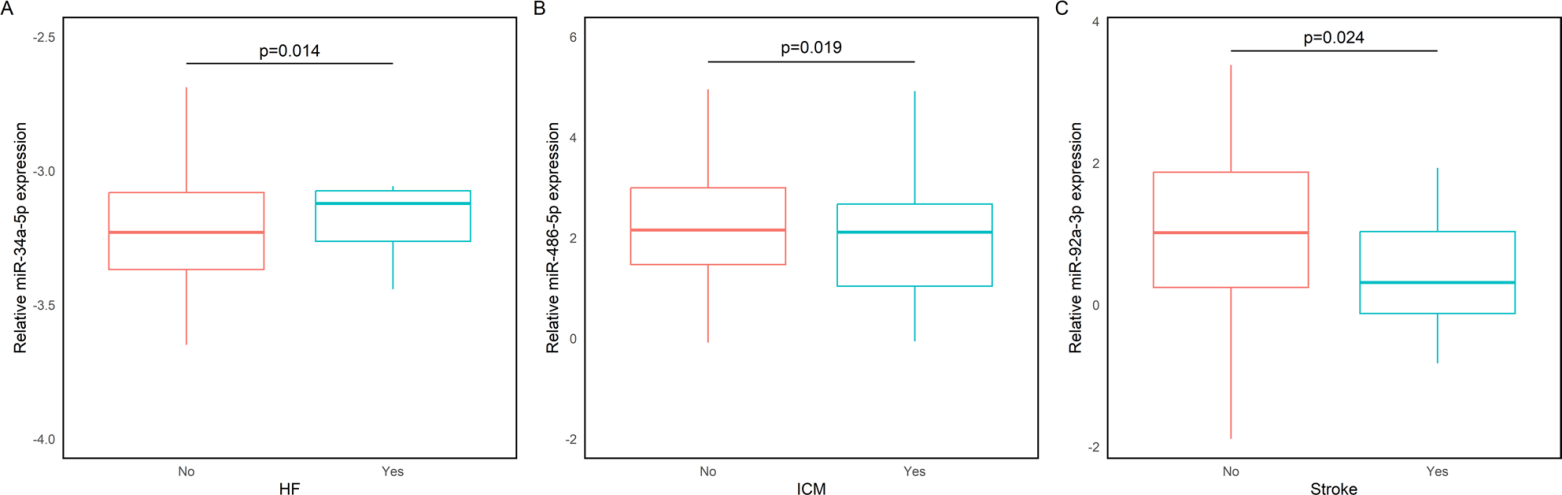
Relative expression levels of miRNAs in patients with and without cardiovascular outcomes. *Note*: Box plots illustrating the relative expression levels of miR-34a-5p (**A)**, miR-486-5p (**B**), and miR-92a-3p (**C**) in patients categorized by the presence (“Yes”) or absence (“No”) of the following conditions: (**A**) Heart Failure (HF), (**B**) Ischemic Cardiomyopathy (ICM), (**C**) Stroke Data are reported as log 2^-ΔCt using miR-484 as endogenous control for normalization *p*-value indicates the significance of the difference after adjusting for age and sex

We then evaluated whether the levels of the selected miRNAs were associated with a diagnosis of CVD. No statistically significant differences were observed between CVD and control groups after adjusting for age and sex (all *p* > 0.05). However, when individual cardiovascular conditions were examined, several miRNAs showed nominal differences in analyses adjusted for age and sex (Fig. 3). Specifically, patients with HF tended to exhibit higher miR-34a-5p expression (Fig. 3A), while lower levels of miR-486-5p were observed in patients with ICM (Fig. 3B), and reduced miR-92a-3p levels were seen in those with troke (Fig. 3C).

Four logistic regression models were applied for each cardiovascular outcome to capture both independent and combined effects of miRNAs and LTL. Models 1–3 were run separately for each miRNA, progressively adjusting for potential confounders. Model 1 included age, sex, and the individual miRNA; Model 2 additionally incorporated established cardiovascular risk factors (BMI, hypertension, diabetes mellitus, LDL-cholesterol, HDL-cholesterol, triglycerides, and creatinine); Model 3 further LTL together with the same single miRNA; and Model 4 integrated all miRNAs jointly with covariates and LTL.

The results of only statistically significant associations (*p* < 0.05) involving miRNAs and/or LTL are reported in Table 2.

**Table 2:** Significant associations between miRNA/telomere levels and cardiovascular diseases across four regression models

CVD	Model^*^	Associated variable	OR (CI 95%)	*p*-value	qFDR^***^
Atrial fibrillation	Model 1	-			
	Model 2	-			
	Model 3	Telomere	< 1^**^	< 0.05^**^	0.21
	Model 4	Telomere miR-23a-3p miR-92a-3p	0.07 (0.01–0.65) 0.47 (0.25–0.88) 1.84 (1.02–3.29)	0.019 0.018 0.045	0.20 0.20 0.27
Heart Failure	Model 1	miR-34a-5p	1.81 (1.14–2.88)	0.014	0.21
	Model 2	miR-34a-5p	1.99 (1.05–3.79)	0.035	0.26
	Model 3	miR-34a-5p	2.02 (1.05–3.82)	0.035	0.21
	Model 4	miR-34a-5p	1.93 (0.92–4.03)	0.080	0.32
Ischemic cardiomyopathy	Model 1	miR-486-5p	0.76 (0.57–0.97)	0.019	0.21
	Model 2	miR-486-5p	0.75 (0.57–0.99)	0.042	0.26
	Model 3	miR-486-5p	0.76 (0.57–0.99)	0.042	0.21
	Model 4	miR-486-5p	0.72 (0.51–1.02)	0.056	0.27
Stroke	Model 1	miR-92a-3p	0.62 (0.41–0.94)	0.024	0.21
	Model 2	miR-92a-3p	0.58 (0.37–0.91)	0.019	0.26
	Model 3	miR-92a-3p	0.57 (0.36–0.91)	0.017	0.21
	Model 4	miR-92a-3p	0.49 (0.27–0.92)	0.025	0.20

^*^ Models 1–3 were run separately for each miRNA. Model 1: age, sex, individual miRNA. Model 2: Model 1 + BMI, hypertension, diabetes, LDL-C, HDL-C, triglycerides, creatinine. Model 3: Model 2 + leukocyte telomere length (LTL) (single miRNA + LTL). Model 4: all miRNAs together + covariates + LTL. Only statistically significant associations (*p* < 0.05) are reported

^**^LTL remained significantly associated with AF in Model 3 (OR < 1 and *p* < 0.05 across all single-miRNA models)

^***^qFDR calculated within each model across all biomarker–outcome tests shown

For AF, the inverse association between LTL and AF risk was maintained across all single-miRNA models (Model 3) and in the fully adjusted Model 4, corroborating previous findings. Notably, in the fully adjusted Model 4, higher miR-23a-3p levels were also inversely associated with AF risk (OR = 0.47, 95% CI: 0.25–0.88, *p* = 0.018), while miR-92a-3p showed a positive association (OR = 1.84, 95% CI: 1.02–3.29, *p* = 0.045), both independently of LTL. Collinearity diagnostics for this model confirmed the stability of these estimates, with Variance Inflation Factor (VIF) values for all predictors below 2.5 (specifically 2.196 for miR-23a-3p and 1.794 for miR-92a-3p) and condition indices within acceptable ranges.

Regarding other disease subtypes, higher miR-34a-5p levels were associated with increased odds of HF in Models 1–3 (OR range: 1.81–2.02; *p* = 0.014–0.035), but this association was no longer statistically significant in Model 4 (OR = 1.93, 95% CI: 0.92–4.03, *p* = 0.080).

Similarly, lower miR-486-5p levels were associated with increased risk of ICM in Models 1–3 (OR range: 0.75–0.76; *p* = 0.019–0.042), with attenuation in Model 4 (OR = 0.72, 95% CI: 0.51–1.02, *p* = 0.056).

For stroke, lower miR-92a-3p levels were consistently associated with increased risk across all four models (OR range: 0.49–0.62; *p* = 0.017–0.025).

Results were unchanged after additional adjustment for inflammatory biomarkers (CRP and albumin) (data not shown).

When accounting for multiple testing, we applied the FDR correction to the full set of biomarker–outcome associations reported in Table 2, within each regression model. After FDR adjustment, none of the miRNA associations reached the q < 0.05 threshold. Corresponding qFDR values are reported in Table 2.

### Predictive performance

**Fig. 4:**
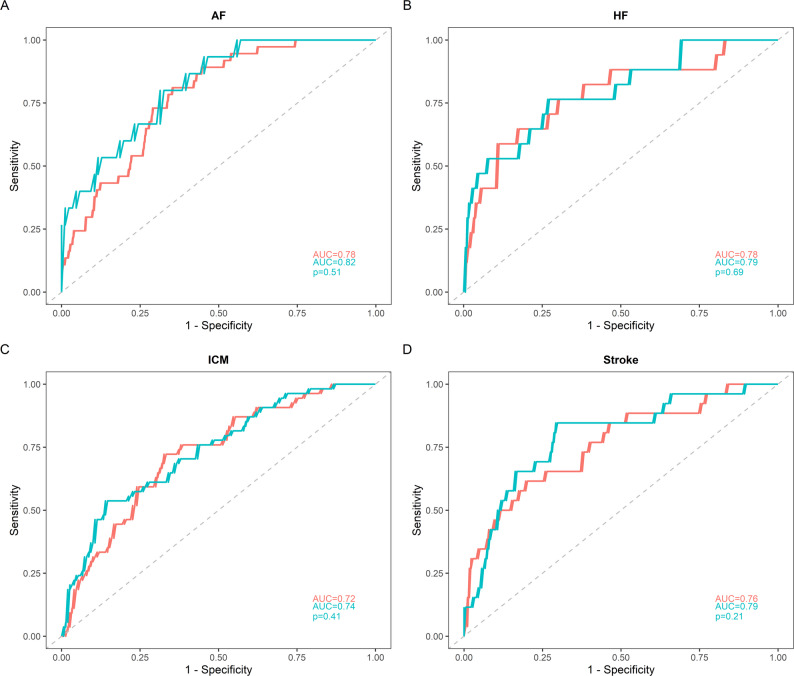
Comparative Performance of Predictive Risk Models for Cardiovascular outcomes. *Note*: The panels display the ROC curves for the different clinical conditions analyzed: (**A**) Atrial Fibrillation (AF), (**B**) Heart Failure (HF), (**C**) Ischemic Cardiomyopathy (ICM), and (**D**) Stroke. The Red curve represents the predictive model based on age, sex, and traditional cardiovascular risk factors (BMI, hypertension, diabetes, HDL cholesterol, LDL cholesterol, triglycerides, and creatinine). The Blue curve represents the predictive model that incorporates an additional biomarker into the traditional model: LTL, miR-23a-3p and miR-92a-3p (**A**), miR-34a-5p (**B**), miR-486-5p (**C**) and miR-92a-3p (**D**). The Area Under the Curve (AUC) values for both models and the *p*-value for the comparison between the two curves are reported for each panel

We evaluated whether the inclusion of telomere length and/or miRNAs could improve the predictive performance of multivariable models for cardiovascular outcomes. Receiver operating characteristic (ROC) curve analyses showed that adding LTL and miR-23a-3p and miR-92a-3p to a model including age, sex, and traditional risk factors marginally increased the AUC for AF from 0.77 to 0.82. Similarly, the addition of miR-34a-5p for HF (AUC from 0.78 to 0.79), miR-486-5p for ICM (AUC from 0.72 to 0.74), and miR-92a-3p for stroke (AUC from 0.76 to 0.79) resulted in only modest improvements. None of these differences reached statistical significance according to DeLong’s test (all *p* > 0.20). These results indicate that telomere length and miRNAs did not substantially improve risk discrimination beyond established clinical predictors (Fig. 4a-d).

## Discussion

Telomere erosion is a physiological hallmark of aging that contributes to individual susceptibility to a wide spectrum of diseases, including cardiovascular disorders [48]. MicroRNAs (miRNAs), in turn, have emerged as modulators of telomere dynamics through the regulation of telomere- and telomerase-associated genes [20, 21] and crucially related to cardiovascular remodelling [14, 15].

In this study, we explored the interplay between leukocyte telomere length (LTL) and circulating levels of five candidate miRNAs, selected for their potential role in telomere biology and cardiovascular risk, in relation to distinct cardiovascular disease (CVD) conditions in an elderly cohort.

Three main findings emerged. First, LTL was not associated with overall CVD status but showed a strong and independent association with atrial fibrillation (AF), with shorter telomeres conferring a markedly increased risk. Second, distinct miRNAs displayed disease-specific associations. Third, no correlations were observed between LTL and the levels of the miRNAs analysed, suggesting that these miRNAs do not mediate the effects of telomere attrition on cardiovascular risk in this cohort.

The robust inverse association between LTL and AF, consistent across multivariable analyses and subsamples, supports prior evidence linking telomere shortening to increased arrhythmic risk [49–52], despite some studies yielding conflicting evidence [53–55]. The specific association between LTL with AF, and the absence of a clear links with other cardiovascular conditions, suggests that telomere shortening may play a distinctive role in atrial vulnerability rather than acting as a general marker for cardiovascular disease.

Mechanistically, telomere-driven cellular senescence promotes atrial vulnerability by impairing calcium homeostasis and compromising mitochondrial function. This leads to subsequent oxidative stress and chronic inflammation, collectively driving the structural and electrical remodelling that facilitates AF [56, 57]. This is supported by recent evidence that telomerase reverse transcriptase (TERT) maintains Ca2 + homeostasis and mitochondrial integrity, with its deficiency promoting arrhythmogenic remodelling [58].

Inflammation represents a plausible biological link between telomere attrition and cardiovascular disease, particularly in older individuals. Chronic low-grade inflammation can accelerate telomere shortening through oxidative stress and increased cellular turnover, while telomere dysfunction may in turn promote a pro-inflammatory state [59]. Consistent with this framework, shorter LTL in our study was associated with adverse inflammatory markers, including higher C-reactive protein and lower albumin levels, suggesting that LTL reflects cumulative inflammatory burden and biological aging. We also observed a negative correlation between LTL and age, consistent with previous reports.

Inflammatory processes may also influence circulating miRNA profiles, as several of the miRNAs investigated have been implicated in immune and endothelial signaling pathways relevant to cardiovascular remodeling [60]. In this context, inflammation may represent a shared biological pathway through which both telomere erosion and miRNA dysregulation contribute to cardiovascular risk. However, we acknowledge that inflammatory status may also capture broader health status (e.g., frailty/comorbidity) and could therefore contribute to residual confounding in observational analyses. Notably, in sensitivity analyses further adjusting for CRP and albumin, the associations were materially unchanged, supporting the robustness of our findings.

Beyond LTL, our findings also implicate specific miRNAs in AF pathogenesis. Individually, miR-23a-3p and miR-92a-3p were not linked to AF; however, in the fully adjusted model, both showed significant associations with AF risk. Notably, they exhibited opposing directions of effect: elevated miR-23a-3p levels were associated with a reduced risk of AF, whereas elevated miR-92a-3p levels were associated with increased risk. These results suggest a potential synergistic or interacting role of these miRNAs in AF development, highlighting that their combined effects may be more biologically relevant than their individual contributions. Furthermore, these associations were independent of LTL, suggesting that miR-23a-3p and miR-92a-3p may influence atrial vulnerability through distinct and complementary molecular mechanisms. Experimental evidence suggests that miR-23a-3p may exert cardioprotective functions, such as attenuating hypertrophic signaling and apoptosis, which could confer resistance against arrhythmic remodelling [61, 62]. Lower levels of miR-23a-3p are also found to be linked to post-operative AF development [63]. Conversely, miR-92a-3p has been implicated in endothelial dysfunction, inflammation, and maladaptive remodelling, all processes that could increase arrhythmic susceptibility [37, 38, 64].

Despite these mechanistic insights, the assessment of their predictive utility showed that combining LTL with miR-23a-3p and miR-92a-3p provided only limited incremental value for AF risk prediction beyond established clinical factors.

Unlike in AF, lower miR-92a-3p levels were associated with an increased risk of stroke, consistent with previous reports of miR-92a downregulation in ischemic stroke patients [65, 66]. Notably, as a member of the miR-17 ~ 92 cluster, miR-92a-3p exerts pleiotropic effects on various cellular and molecular pathways, including neurogenesis, neural repair, and neuroinflammation, processes central to stroke pathology and recovery [67].

Beyond these associations, disease-specific patterns were observed for other miRNAs. Elevated miR-34a-5p levels were associated with increased odds of HF in our dataset, consistent with its established role in diverse cardiac biological pathways implicated in cardiovascular injury and dysfunction, as reviewed by [34]. Specifically, miR-34a overexpression has been linked to impaired autophagy, apoptosis, inflammation, fibrosis, remodelling, and cardiac aging, while its inhibition has been reported to confer cardioprotective effects and improve cardiac function [34 and references therein].

Our study also revealed an association between lower levels of miR-486-5p and an increased risk of ICM. Consistent with this, experimental studies have shown that overexpression of miR-486-5p protects against ischemic injury and cardiomyocyte apoptosis through multiple signaling pathways [68–71] and plays an important role in inhibiting myocardial fibrosis [39]. Moreover, miR-486 dysregulation has been observed in coronary atherosclerotic plaques [72], suggesting a broader contribution to both myocardial vulnerability and vascular pathology.

The association between miR-486-5p and ICM observed in our study could partially be related to its correlation with inflammatory status, as evidenced by its significant correlation with low levels of albumin.

Nevertheless, despite these disease-specific associations, the addition of these miRNAs to models including conventional clinical predictors did not substantially improve discriminatory performance, indicating limited utility as biomarkers for risk prediction.

One of the notable findings of our analysis was the absence of significant correlations between LTL and the circulating levels of the five candidate miRNAs. Some of these miRNAs, including miR-21-5p and miR-34a-5p, which regulate TERT and telomerase activity [27, 35], and miR-23a-3p, which targets the telomere-protective protein TRF2 [32], are known to influence telomere biology, leading us to hypothesize a possible interconnection in cardiovascular disease. However, our results suggest that plasma levels of these miRNAs may not directly mirror LTL and that the two reflect distinct biological pathways. While telomere shortening primarily captures cellular senescence and mitochondrial decline, the circulating miRNAs assessed here appear to reflect inflammatory, endothelial, and remodelling processes. This lack of correlation indicates that LTL and these miRNAs may contribute to AF and other CVD phenotypes through complementary but separate mechanisms, even if their combined predictive utility is limited.

Potential reasons for the lack of correlation may include that intracellular miRNA functions do not necessarily mirror their extracellular levels, which depend on secretion and release mechanisms, as well as tissue-specific regulation or the limited statistical power of our subset.

We acknowledge several limitations. The subset with miRNA measurements was smaller, which may have reduced statistical power, and although some baseline differences were observed, the overall demographic and clinical profile remained broadly comparable to that of the full cohort, suggesting that major selection bias is unlikely. Only five candidate miRNAs were analysed, chosen for their relevance to both cardiovascular disease and telomere biology. The study population consisted of elderly individuals, which may limit generalizability to younger or more diverse groups. Furthermore, after correction for multiple testing using a global false discovery rate (FDR) approach, the associations involving circulating miRNAs did not retain formal statistical significance. Given that our analyses were hypothesis-driven and focused on a limited set of biologically relevant miRNAs rather than on an exploratory screening, these findings should be interpreted as hypothesis-generating. This interpretation is supported by the internal consistency of effect estimates across different levels of adjustment and by the stability of the multivariable models, as confirmed by collinearity diagnostics. Finally, although LTL and miRNAs were associated with cardiovascular outcomes, their addition to traditional risk factors did not substantially improve predictive accuracy.

Despite these limitations, the study has notable strengths, including a well-characterized cohort, simultaneous assessment of LTL and circulating miRNAs, and evaluation across multiple CVD phenotypes.

## Conclusions

Our findings suggest that telomere erosion and miRNA dysregulation contribute to cardiovascular pathology through complementary but independent mechanisms, with shorter telomeres linked to AF and distinct miRNAs associated with other CVD outcomes. These results extend previous evidence connecting telomere attrition and miRNA imbalance to cardiovascular aging and highlight possible disease-specific molecular signatures. Although the addition of these biomarkers did not substantially improve risk prediction, they may still provide valuable mechanistic insights into how biological aging influences cardiovascular vulnerability in older adults. Future studies in larger and more diverse elderly cohorts, ideally with longitudinal follow-up, are warranted to clarify causal relationships and explore the combined utility of LTL, miRNAs, and other biomarkers for risk stratification in aging populations.

## Supplementary Information

Supplementary Material 1.

Supplementary Material 2.

Supplementary Material 3.

## Data Availability

The datasets generated and analysed during the current study are not publicly available due to institutional and ethical restrictions related to the privacy of Nursing Home Residents. However, de-identified data may be made available from the corresponding author upon reasonable request and with appropriate approval from the Institutional Review Board or relevant Ethics Committee.

## Abbreviations

AF: Atrial Fibrillation
AUC: Area Under the Curve
BMI: Body Mass Index
cDNA: Complementary DNA
CI: Confidence Interval
CVD: Cardiovascular Disease
DBP: Diastolic Blood Pressure
HbA1C: Glycated Haemoglobin
HDL: High-Density Lipoprotein
HF: Heart Failure
ICM: Ischemic Cardiomyopathy
LDL: Low-Density Lipoprotein
LTL: Leukocyte telomere length
miRNA: microRNA
OR: Odds Ratios
qPCR: Quantitative Polymerase Chain Reaction
ROC: Receiver Operating Characteristic
SBP: Systolic Blood Pressure
SD: Standard Deviation
TERT: Telomerase Reverse Transcriptase
TL: Telomere Length
TRF2: Telomeric Repeat Binding Factor 2
