# Supplementary material for: Leukocyte telomere length and circulating MiRNAs in relation to cardiovascular outcomes in older adults
Source: BMC Geriatr. 2026 Feb 2;26:292. doi: 10.1186/s12877-026-07042-4 (PMC12955057; doi:10.1186/s12877-026-07042-4)
Supplement: Supplementary file 1 — Supplementary Material 1. [file 12877_2026_7042_MOESM1_ESM.pdf]

Supplementary File 1: Questionnaire

English version of the structured questionnaire developed for this study and approved by the local Ethics Committee. The questionnaire was used to collect information on participants' sociodemographic characteristics, medical history, medication use, lifestyle habits, and functional status. It was administered by trained personnel during the baseline assessment in Nursing Homes.

Personal Data Sheet

Patient Code	☐☐☐☐☐☐☐
Date of Visit	________
Last Name	________
First Name	________
Sex / Gender	M F
City of Birth	________
Date of Birth	________
City of Residence	________
Address	________
Phone Number	________
Fiscal Code	________

Cohabitation

1. Spouse ☐
2. Sisters/Brothers ☐
3. Son/Daughter ☐
4. Other Relatives ☐
5. Close Friend(s) ☐
6. Nursing Home ☐

Your residence is:

1. Apartment ☐
2. Home for the Elderly ☐
3. Nursing Home ☐
4. Other: ☐

Marital Status:

1. Single (Unmarried) ☐
2. Married ☐
3. Widowed ☐
4. Separated ☐

Educational Level

Never attended school ☐
Incomplete primary school ☐
Primary school diploma ☐
Middle school diploma ☐
High school diploma ☐
Degree (University) ☐
Illiterate ☐

Economic Resources

Very poor ☐
Fairly poor ☐
Good ☐
Very good ☐
Excellent ☐

Place of Interview________________________

Cognitive and Functional Status

**Mini-Mental State Examination**	
What year is it? (0-1)	
What season is it? (0-1)	
What month is it? (0-1)	
What is today's date? (0-1)	
What day of the week is it today? (0-1)	
What country are we in? (0-1)	
What Italian region are we in? (0-1)	
What city are we in? (0-1)	
What is the name of the place we are in? (0-1)	
What floor are we on? (0-1)	
Have them repeat: "House, bread, cat" (0-3)	
Have them count backwards from 100, subtracting 7 five times (93-86-79-72-65). Alternatively, have them spell the word WORLD backwards (D-L-R-O-W) (0-5)	
Ask for the repetition of the 3 names learned previously (0-3)	
Show a pencil and a watch asking for their names (0-2)	
Ask them to repeat the sentence: "Tiger against tiger" (0-1)	
Take a sheet of paper, fold it in half, and place it on the table (0-3)	
Execute the written command (0-1) (0-1)<br><br>CLOSE YOUR EYES	
**Write a sentence (must contain subject and verb) (0-1)**<br>________________	
**Copy the drawing (0-1)**<br>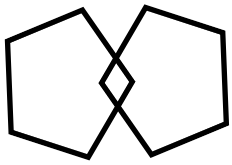	
**Total MMSE Score**	
**Test not administrable**	

Basic Activities of Daily Living (ADL)

Feeding

Does he/she usually manage to feed himself/herself completely and without difficulty? Yes / No

How long has he/she required assistance with feeding?

1. Less than one year
2. Several years (less than ten)
3. More than ten years

Mobility

Does he/she usually get in and out of bed completely by himself/herself and without any difficulty? Yes / No

How long has he/she required assistance with mobility?

1. Less than one year
2. Several years (less than ten)
3. More than ten years

Dressing and Undressing

Does he/she usually dress and undress completely by himself/herself and without any difficulty? Yes / No

How long has he/she required assistance with dressing?

1. Less than one year
2. Several years (less than ten)
3. More than ten years

Toilet Use

Does he/she usually use the toilet completely by himself/herself and without difficulty? Yes / No

How long has he/she required assistance with using the toilet?

1. Less than one year
2. Several years (less than ten)
3. More than ten years

Showering and Bathing

Does he/she usually shower or bathe completely by himself/herself and without difficulty? Yes / No

How long has he/she required assistance with washing?

1. Less than one year
2. Several years (less than ten)
3. More than ten years

Incontinence

Does he/she usually manage to control feces and urine? Yes / No

Does he/she use catheters and/or diapers? Yes / No

Da quanto tempo è assistito?

1. Less than one year
2. Several years (less than ten)
3. More than ten years

Instrumental Activities of Daily Living (IADLs)

**Do you usually prepare meals completely by yourself and without any difficulty?**	
1. Yes, without any difficulty and completely by myself	
2. No, with some difficulty, but completely by myself	
3. No, not completely by myself (go to the next question)	
4. I do not prepare meals (go to the next question)	
5. If you have to or want to, can you do it alone without any difficulty?	Yes / No

**Do you usually do light, routine housework completely by yourself and without any difficulty?**	
1. Yes, without any difficulty and completely by myself	
2. No, with some difficulty, but completely by myself	
3. No, not completely by myself (go to the next question)	
4. I do not do light housework (go to the next question)	
5. If you have to or want to, can you do it alone without any difficulty?	Yes / No

**Do you usually do heavy, periodic housework completely by yourself and without any difficulty?**	
1. Yes, without any difficulty and completely by myself	
2. No, with some difficulty, but completely by myself	
3. No, not completely by myself (go to the next question)	
4. I do not do heavy housework (go to the next question)	

5. If you have to or want to, can you do it alone without any difficulty?	Yes	No

Do you usually go to do general shopping completely by yourself and without any difficulty?	
1. Yes, without any difficulty and completely by myself	
2. No, with some difficulty, but completely by myself	
3. No, not completely by myself (go to the next question)	
4. I do not go shopping (go to the next question)	
5. If you have to or want to, can you do it alone without any difficulty?	Yes / No

Do you usually do the laundry completely by yourself and without any difficulty?	
1. Yes, without any difficulty and completely by myself	
2. No, with some difficulty, but completely by myself	
3. No, not completely by myself (go to the next question)	
4. I do not do the laundry (go to the next question)	
5. If you have to or want to, can you do it alone without any difficulty?	Yes / No

Do you usually use the telephone completely by yourself and without any difficulty?	
1. Yes, without any difficulty and completely by myself	
2. No, with some difficulty, but completely by myself	
3. No, not completely by myself (go to the next question)	
4. I do not use the telephone (go to the next question)	
5. If you have to or want to, can you do it alone without any difficulty?	Yes / No

Do you usually take your medication completely by yourself and without any difficulty?	
1. Yes, without any difficulty and completely by myself	
2. No, with some difficulty, but completely by myself	
3. No, not completely by myself (go to the next question)	
4. I do not take medication (go to the next question)	

5. If you have to or want to, can you do it alone without any difficulty?	Yes	No

Do you usually manage your financial matters completely by yourself and without any difficulty?	
1. Yes, without any difficulty and completely by myself	
2. No, with some difficulty, but completely by myself	
3. No, not completely by myself (go to the next question)	
4. I do not manage financial matters (go to the next question)	
5. If you have to or want to, can you do it alone without any difficulty?	Yes / No

Smoking and Alcohol Habits

Smoker	
Ex-smoker	
Never smoked	
How many cigarettes do you smoke on average per day?	
For how many years did you smoke regularly?	

Do you currently consume alcoholic beverages?	
If yes, how many glasses per week?	
Did you consume alcohol previously?	
If yes, how many glasses per week?	

Health Status and Morbidity

How would you define your general state of health?

1. Excellent	
2. Very Good	
3. Good	
4. Distressed	
5. Very Distressed	

Have you ever fallen in the last year? Yes No

If yes, how many times

**In the last year, how many times have you been hospitalized?**				
**In the last year, have you sustained any fractures?**		**Yes**		**No**
**Location of the fracture**				

Cardiovascular Pathologies

1. Arterial Hypertension	
2. Ischemic Heart Disease	
3. TSA Atheromatosis	
4. Pulmonary Embolism	
5. Atrial Fibrillation	
6. Deep Vein Thrombosis	
7. Orthostatic Hypotension	
8. Peripheral Vascular Disease	
9. Claudication	
10. Heart Failure	
**Classe NYHA**	

Neurological Pathologies

1. T.I.A.	
2. Stroke	
3. Hemiparesis/Hemiplegia	
4. Non-Alzheimer's Dementia	
5. Tetraplegia	
6. Parkinson's Disease	
7. Alzheimer's Disease	
8. Epilepsy	

Gastrointestinal Pathologies

1. Gastritis	
2. Ulcer	
3. Dyspepsia	
4. Constipation	
5. Diarrhea	
6. Alternating Bowel Habits	
7. Diverticulosis/Diverticulitis	
8. Colon Polyposis	

Endocrine Pathologies

1. Diabetes Mellitus	
2. Subclinical Hypothyroidism	
3. Clinical Hypothyroidism	
4. Subclinical Hyperthyroidism	
5. Clinical Hyperthyroidism	

Musculoskeletal Pathologies

1. Osteoarthritis	
2. Rheumatoid Arthritis	
3. Osteoporosis	

Pulmonary Pathologies

1. Asthma	
2. Emphysema	
3. COPD	

Sensory Organ Pathologies

1. Glaucoma	
2. Deafness	
3. Macular Degeneration	
4. Cataract	

Genitourinary Pathologies

1. Urinary Tract Infection	
2. Dialysis	
3. Prostatic Hypertrophy	
4. Renal Failure	

Infectious Pathologies

1. Fever (currently present)	
2. Infections	
3. Septic Shock	

Neoplastic Pathologies

1. Non-metastatic Tumor	
2. Metastatic Tumor	

**Cumulative Illness Rating Scale (CIRS)**					
**Organ Systems**	**rating**				
Cardiac (Heart only)	**0**	**1**	**2**	**3**	**4**
Arterial Hypertension (Severity rating; organ damage is rated separately)	**0**	**1**	**2**	**3**	**4**
Vascular (Blood, blood vessels, and blood cells, bone marrow, spleen, lymph nodes)	**0**	**1**	**2**	**3**	**4**
Respiratory System (Lungs, bronchi, trachea below the larynx)	**0**	**1**	**2**	**3**	**4**
Eyes, Ears, Nose, Pharynx, Larynx	**0**	**1**	**2**	**3**	**4**
Upper Gastrointestinal System (Esophagus, stomach and duodenum; pancreas; excluding diabetes)	**0**	**1**	**2**	**3**	**4**
Lower Gastrointestinal System (Intestines, hernias)	**0**	**1**	**2**	**3**	**4**
Hepatic (Liver and bile ducts)	**0**	**1**	**2**	**3**	**4**
Renal (Kidneys only)	**0**	**1**	**2**	**3**	**4**
Genitourinary System (Ureters, bladder, urethra, prostate, genital system)	**0**	**1**	**2**	**3**	**4**
Musculoskeletal System and Skin (Muscles, skeleton, integuments)	**0**	**1**	**2**	**3**	**5**
Central and Peripheral Nervous System (Brain, spinal cord, nerves; excluding dementia)	**0**	**1**	**2**	**3**	**4**
Endocrine-Metabolic System (Including diabetes, thyroid; breast; systemic infections; intoxications)	**0**	**1**	**2**	**3**	**4**
Psychiatric / Behavioral Disorders (Including dementia, depression, anxiety, agitation/delirium; psychosis)	**0**	**1**	**2**	**3**	**4**

Medications

For each medication, please list:
Commercial Name
Dose
Days/Week (D/W)
Medical Prescription (MP)
Sporadic Use (SU) / Taken as Needed
If the use of the medication has been discussed with the patient (DP)/family members (DF)

Name	Dose	D/W	MP	SU	DP/DF

Geriatric Depression Scale

	Yes	No
Are you basically satisfied with your life?	0	1
Have you dropped many of your activities and interests?	1	0
Does your life feel empty?	1	0
Are you often bored?	1	0
Are you in good spirits most of the time?	0	1
Are you afraid that something bad is going to happen to you?	1	0
Do you feel happy most of the time?	0	1
Do you often feel helpless?	1	0
Do you prefer to stay at home rather than going out and doing new things?	1	0
Do you feel you have more memory problems than most?	1	0
Do you think it is wonderful to be alive now?	0	1
Do you feel worthless the way you are now?	1	0
Do you feel full of energy?	0	1
Do you feel that your situation is hopeless?	1	0
Do you think that most people are better off than you are?	1	0

GDS Total Score ☐

Test not administrable ☐

CES-D Fatigue Questions

How often during the past week did you feel that everything you did was an effort?

1. Rarely or none of the time ☐
2. Some of the time ☐
3. Occasionally ☐
4. Most or all of the time ☐

How often during the past week did you feel you couldn't keep going?

1. Rarely or none of the time ☐
2. Some of the time ☐
3. Occasionally ☐
4. Most or all of the time ☐

Anthropometric Measures

1. Height ☐
2. Knee Height ☐
3. Weight ☐
4. Weight loss in the last yea ☐
5. Biceps Circumference ☐

Blood Pressure and Heart Rate

	SBP	DBP	HR
Measurement 1			
Measurement 2			
Measurement 3			

Physical Function Tests

Grip Strength

Dominant Hand — Right ☐ Left ☐

Position — Sitting ☐ In bed ☐

Measurement 1 Right ☐
Measurement 1 Left ☐

Measurement 2 Right ☐
Measurement 2 Left ☐
Test not performable ☐

Gait Speed over 4 meters (seconds)

Measurement 1 ☐
Measurement 2 ☐
Test not performable ☐

Chair Stand Test 5 consecutive times (seconds)

Measurement ☐
Test not performable ☐

Balance

0. Unable to hold feet side-by-side standing position for 10 seconds	
1. Able to hold feet side-by-side for 10 sec, but not semi-tandem	
2. Able to hold semi-tandem position for 10 sec, but not tandem	
3. Able to hold tandem position for more than 2 sec, but less than 10 sec	
4. Able to hold tandem position for more than 10 sec	
**Score**	

Metabolic Holter

Total Caloric Expenditure	
Active Caloric Expenditure	
Resting Caloric Expenditure	
Physical Activity Intensity (METs)	
Duration of Physical Activity	
Total Number of Steps	
Sleep Duration	
Time Spent Lying Down	

Bioelectrical Impedance Analysis (BIA) Parameters

Resistance	
Reactance	
Angolo di fase	
Lean Mass (Kg)	
Lean Mass (%)	
Fat Mass (Kg)	
Fat Mass (%)	
Muscle Mass (Kg)	
Muscle Mass (%)	
Cell Mass (Kg)	
Cell Mass (%)	
Total Water (l)	
Total Water (%)	
Extracellular Water (l)	
Extracellular Water (%)	
Intracellular Water (l)	
Intracellular Water (%)	
BCMI (Body Cell Mass Index)	

Test not administrable ☐
Reason:

Laboratory Parameters

**Renal Function Test**	
Azotaemia	
Creatinine	
Uric Acid	
**Liver Function Tests**	
AST - Aspartate Aminotransferase	
ALT - Alanine Aminotransferase	
Gamma-Glutamyl Transferase	
Alkaline Phosphatase	
Total Bilirubin	
Direct Bilirubin	
**Lipid Panel Tests**	
Total Cholesterol	
HDL Cholesterol	
LDL Cholesterol	
LDL Cholesterol	
**Nutritional Profile Tests**	
Blood Glucose	
Glycated Hemoglobin (HbA1c)	
Total Proteins	
Albumin	
Prealbumina	
**Electrolyte Balance**	
Sodium	
Potassium	
Chloride	
Calcium	
Phosphorus	
Magnesium	
**Complete Blood Count**	
Red Blood Cells	
White Blood Cell	
Neutrophils	
Eosinophils	
Basophils	
Lymphocytes	
Monocytes	
Platelets	
Hemoglobin	
Hematocrit	
Mean Corpuscular Volume (MCV)	
Mean Corpuscular Hemoglobin (MCH)	
Mean Corpuscular Hemoglobin Concentration (MCHC)	
Red Cell Distribution Width (RDW)	
Mean Platelet Volume (MPV)	
Serum iron	
Ferritin	
C-reactive protein (CRP)	
**Urine Analysis**	
Appearance	
Color	
pH	
Density	
Proteinuria	
Hematuria	
Leukocytes	
Nitrites	
Bilirubin	
Urobilinogen	
Glucose	
Ketones	

Long MNA®
Mini Nutritional Assessment

Last name: ______ First name: ______

Sex: ______ Age: ______ Weight, kg: ______ Height, cm: ______ Date: ______

Complete the screen by filling in the boxes with the appropriate numbers.
Add the numbers for the screen. If score is 11 or less, continue with the assessment to gain a Malnutrition Indicator Score.

Screening

A Has food intake declined over the past 3 months due to loss of appetite, digestive problems, chewing or swallowing difficulties?
0 = severe decrease in food intake
1 = moderate decrease in food intake
2 = no decrease in food intake ☐

B Weight loss during the last 3 months
0 = weight loss greater than 3kg (6.6lbs)
1 = does not know
2 = weight loss between 1 and 3kg (2.2 and 6.6 lbs)
3 = no weight loss ☐

C Mobility
0 = bed or chair bound
1 = able to get out of bed / chair but does not go out
2 = goes out ☐

D Has suffered psychological stress or acute disease in the past 3 months?
0 = yes 2 = no ☐

E Neuropsychological problems
0 = severe dementia or depression
1 = mild dementia
2 = no psychological problems ☐

F Body Mass Index (BMI) = weight in kg / (height in m)^2
0 = BMI less than 19
1 = BMI 19 to less than 21
2 = BMI 21 to less than 23
3 = BMI 23 or greater ☐

Screening score (subtotal max. 14 points) ☐☐

12-14 points:	Normal nutritional status
8-11 points:	At risk of malnutrition
0-7 points:	Malnourished

For a more in-depth assessment, continue with questions G-R

Assessment

G Lives independently (not in nursing home or hospital)
1 = yes 0 = no ☐

H Takes more than 3 prescription drugs per day
0 = yes 1 = no ☐

I Pressure sores or skin ulcers
0 = yes 1 = no ☐

J How many full meals does the patient eat daily?
0 = 1 meal
1 = 2 meals
2 = 3 meals ☐

K Selected consumption markers for protein intake
- At least one serving of dairy products (milk, cheese, yoghurt) per day yes ☐ no ☐
- Two or more servings of legumes or eggs per week yes ☐ no ☐
- Meat, fish or poultry every day yes ☐ no ☐

0.0 = if 0 or 1 yes
0.5 = if 2 yes
1.0 = if 3 yes ☐.☐

L Consumes two or more servings of fruit or vegetables per day?
0 = no 1 = yes ☐

M How much fluid (water, juice, coffee, tea, milk...) is consumed per day?
0.0 = less than 3 cups
0.5 = 3 to 5 cups
1.0 = more than 5 cups ☐.☐

N Mode of feeding
0 = unable to eat without assistance
1 = self-fed with some difficulty
2 = self-fed without any problem ☐

O Self view of nutritional status
0 = views self as being malnourished
1 = is uncertain of nutritional state
2 = views self as having no nutritional problem ☐

P In comparison with other people of the same age, how does the patient consider his / her health status?
0.0 = not as good
0.5 = does not know
1.0 = as good
2.0 = better ☐.☐

Q Mid-arm circumference (MAC) in cm
0.0 = MAC less than 21
0.5 = MAC 21 to 22
1.0 = MAC greater than 22 ☐.☐

R Calf circumference (CC) in cm
0 = CC less than 31
1 = CC 31 or greater ☐

Assessment (max. 16 points) ☐☐.☐
Screening score ☐☐.☐
Total Assessment (max. 30 points) ☐☐.☐

Malnutrition Indicator Score

24 to 30 points	☐	Normal nutritional status
17 to 23.5 points	☐	At risk of malnutrition
Less than 17 points	☐	Malnourished

References
1. Vellas B, Villars H, Abellan G, *et al.* Overview of the MNA® - Its History and Challenges. *J Nutr Health Aging*. 2006; **10:456**-465.
2. Rubenstein LZ, Harker JO, Salva A, Guigoz Y, Vellas B. Screening for Undernutrition in Geriatric Practice: Developing the Short-Form Mini Nutritional Assessment (MNA-SF). *J. Geront*. 2001; **56A**: M366-377
3. Guigoz Y. The Mini-Nutritional Assessment (MNA®) Review of the Literature - What does it tell us? *J Nutr Health Aging*. 2006; **10**:466-487.

Registered trademark of Société des Produits Nestlé S.A. © Société des Produits Nestlé S.A “amended 2023”.
