# Supplementary material for: Leukocyte telomere length and circulating MiRNAs in relation to cardiovascular outcomes in older adults
Source: BMC Geriatr. 2026 Feb 2;26:292. doi: 10.1186/s12877-026-07042-4 (PMC12955057; doi:10.1186/s12877-026-07042-4)
Supplement: Supplementary file 2 — Supplementary Material 2. [file 12877_2026_7042_MOESM2_ESM.docx]

**Supplementary File 2.** Comparison between participants with and without available plasma samples for miRNA analysis.

| **Variable** | **Overall (n=624)** | **No miRNA (n=414)** | **Yes miRNA (n=210)** | **p-value** |
| --- | --- | --- | --- | --- |
| Age, mean (SD) | 81.08 (7.80) | 80.08 (7.80) | 83.69 (7.20) | <0.001 |
| Female sex % | 68.6 | 68.3 | 69.3 | 0.887 |
| BMI, mean (SD) | 27.05 (6.05) | 27.54 (5.91) | 26.89 (5.24) | 0.178 |
| Total cholesterol, mean (SD) | 164.09 (39.81) | 168.23 (39.13) | 162.15 (39.79) | 0.084 |
| HDL cholesterol, mean (SD) | 51.52 (15.95) | 52.10 (16.89) | 49.08 (13.11) | 0.017 |
| LDL cholesterol, mean (SD) | 93.11 (32.46) | 95.65 (31.33) | 87.22 (33.63) | 0.004 |
| Triglycerides, mean (SD) | 122.54 (67.21) | 123.77 (68.04) | 119.50 (65.21) | 0.489 |
| Glucose, mean (SD) | 101.46 (38.29) | 101.93 (40.33) | 100.32 (32.85) | 0.649 |
| HbA1c, mean (SD) | 6.06 (1.46) | 6.04 (1.48) | 6.08 (1.42) | 0.814 |
| Mean DBP, mean (SD) | 73.75 (7.65) | 73.71 (7.48) | 73.85 (8.20) | 0.870 |
| Mean SBP, mean (SD) | 126.41 (12.02) | 126.62 (11.83) | 125.75 (12.63) | 0.496 |
| Azotemia, mean (SD) | 49.06 (24.18) | 49.51 (25.14) | 47.99 (21.76) | 0.503 |
| Creatinine, mean (SD) | 1.10 (0.43) | 1.11 (0.43) | 1.09 (0.44) | 0.596 |
| Uric acid, mean (SD) | 4.82 (1.45) | 4.90 (1.46) | 4.73 (1.40) | 0.177 |
| Albumin, mean (SD) | 54.02 (6.37) | 54.48 (5.87) | 53.89 (6.37) | 0.287 |
| C-reactive protein, mean (SD) | 12.06 (22.98) | 10.58 (22.75) | 14.53 (23.24) | 0.060 |
| CVD yes, % | 37.18 | 31.88 | 47.6 | 0.002 |
| Hypertension yes, % | 72.6 | 72.3 | 73.3 | 0.899 |
| Diabetes mellitus yes, % | 25.8 | 26.5 | 23.8 | 0.556 |
| Atrial fibrillation yes, % | 9.9 | 9.1 | 12.2 | 0.307 |
| Ischemic cardiopaty, yes, % | 22.6 | 21.9 | 24.4 | 0.572 |
| Heart failure yes, % | 7.5 | 7.3 | 8.1 | 0.853 |
| Stroke yes, % | 9.0 | 7.7 | 12.2 | 0.112 |

**Note:** p-values refer to comparisons between participants without and with miRNA data. The “Overall” column is provided for descriptive purposes only.
