# Supplementary material for: Leukocyte telomere length and circulating MiRNAs in relation to cardiovascular outcomes in older adults
Source: BMC Geriatr. 2026 Feb 2;26:292. doi: 10.1186/s12877-026-07042-4 (PMC12955057; doi:10.1186/s12877-026-07042-4)
Supplement: Supplementary file 3 — Supplementary Material 3. [file 12877_2026_7042_MOESM3_ESM.docx]

**Supplementary File 3**: Baseline characteristics of the study population subsample stratified by cardiovascular disease (CVD) status.

|  | **CVD** | | |
| --- | --- | --- | --- |
| **Variable** | **Absent (n = 110)** | **Present (n = 100)** | **p-value** |
| Age (years) | 82.24 ±7.83 | 85.05 ±6.00 | 0.005 |
| Female sex, % | 72.7 | 70.4 | 0.414 |
| BMI (kg/m²) | 24.85 ±5.08 | 24.67 ±5.31 | 0.677 |
| Total Cholesterol (mg/dL) | 161.32 ±40.41 | 144.79 ±38.15 | 0.003 |
| HDL Cholesterol (mg/dL) | 47.49 ±12.78 | 47.15 ±12.05 | 0.687 |
| LDL Cholesterol (mg/dL) | 90.93 ±29.66 | 76.55 ±29.20 | 0.001 |
| Triglycerides (mg/dL) | 119.52 ±67.14 | 115.93 ±58.54 | 0.210 |
| Fasting Glucose (mg/dL) | 101.70 ±36.68 | 99.26 ±30.41 | 0.946 |
| HbA1c (%) | 6.21 ±1.53 | 6.01 ±1.68 | 0.143 |
| DBP mean (mmHg) | 74.08 ±9.03 | 73.00 ±9.47 | 0.510 |
| SBP mean (mmHg) | 126.46 ±12.54 | 124.38 ±13.76 | 0.562 |
| Azotemia (mg/dL) | 46.41 ±18.22 | 55.58 ±30.28 | 0.087 |
| Creatinine (mg/dL) | 1.08 ±0.42 | 1.19 ±0.60 | 0.202 |
| Uric Acid (mg/dL) | 4.45 ±1.34 | 4.83 ±1.55 | 0.115 |
| Albumin (g/L) | 52.60 ±7.14 | 52.76 ±6.80 | 0.870 |
| C-reactive Protein (mg/L) | 13.32 ±21.37 | 16.46 ±33.79 | 0.438 |
| Hypertension, yes (%) | 66.1 % | 77.9 % | 0.086 |
| Diabetes Mellitus, yes, (%) | 25.7 % | 26.3 % |  |
| Atrial Fibrillation, yes (%) |  | 27.4 % |  |
| Ischemic Cardiopaty, yes (%) |  | 56.8 % |  |
| Heart Failure, yes (%) |  | 17.9 % |  |
| Stroke, yes (%) |  | 27.4 % |  |

*Note:* Continuous variables are presented as mean ± SD and categorical variables as number (%).

*P value from t-test or Mann–Whitney depending on continuous data distribution and Fisher’s exact test of association for categorical variables

*Abbreviations*: BMI, Body Mass Index; HDL, High Density Lipoprotein; LDL, Low Density Lipoprotein; HbA1c, Glycated Haemoglobin; SBP, Systolic Blood Pressure; DBP, Diastolic Blood Pressure.
